# Bond Strength and Adhesion Mechanisms of Novel Bone Adhesives

**DOI:** 10.3390/bioengineering10010078

**Published:** 2023-01-06

**Authors:** Sarah J. Upson, Matthew J. Benning, David A. Fulton, Ian P. Corbett, Kenneth W. Dalgarno, Matthew J. German

**Affiliations:** 1 Department of Life and Environmental Sciences, Faculty of Science and Technology, Bournemouth University, Poole BH12 5BB, UK; 2 School of Engineering, Newcastle University, Newcastle upon Tyne NE1 7RU, UK; 3 Chemical Nanoscience Laboratory, Chemistry-School of Natural and Environmental Sciences, Newcastle University, Newcastle upon Tyne NE1 7RU, UK; 4 School of Dental Sciences, Translational and Clinical Research Institute, Faculty of Medical Sciences, Newcastle University, Newcastle upon Tyne NE2 4AZ, UK

**Keywords:** fracture healing, bone adhesive, biocompatibility, methacryloyloxydecyl methacrylate dihydrogen phosphate (mdp/10-mdp), bone repairing, in vitro models for fracture healing

## Abstract

Bone adhesives offer distinct advantages over the use of screws to attached internal fixation plates (IFPs). As the chemical composition of bone is similar to dentine, it is possible that the types of monomers used to make dentine adhesives could be utilised to affix IFPs to bone. The ability to attach a bio-resorbable IFP to porcine bone was assessed for the monomer 10-methacryloyloxydecyl dihydrogen phosphate (MDP), used either as a homopolymer or a copolymer with urethane dimethacrylate (MDP + U). Additionally, the addition of a priming step (MDP + U + P) was evaluated. The chemical interactions of the monomers with bone were assessed using XRD and imaged using TEM, revealing the formation of nano-layered structures with the MDP primer, something we believe has not been reported on bone. In a 6-week artificial aging study both MDP + U and MDP + U + P demonstrated adequate shear bond strength to affix bio-resorbable IFPs. The cytotoxicity profiles of the adhesive formulations were determined using indirect and direct contact with MC3T3 cells, with indirect conditions suggesting the MDP + U + P is as cytocompatible as the resorbable IFP. The findings of this study suggest our newly developed adhesive has the potential to be used as a bone adhesive to affix bioresorbable IFPs.

## 1. Introduction

Cranio-maxillofacial bone fractures are the third most common type of fracture to occur in the UK [1]. Accounting for between 5%–10% of all attendances to accident and emergency departments [2], they most commonly occur as a result of trauma, with falls, road traffic accidents and interpersonal violence the most frequent causes globally [1,3,4]. Due to the complex anatomy of the cranio-maxillofacial region and the need to ingest food, stabilising the fracture via an external fixation/immobilisation is not ideal, and internal fixation plates (IFPs) are the most commonly used method to aid healing. Made of titanium alloys, or resorbable polymers and copolymers of polylactic acid and polyglycolic acid, the IFPs are attached to the bone using screws. However, screw failure rates can be as high as 25% [5,6], screw detachment can lead to post-operative complications for a variety of reasons including; over-tightening of the screws on insertion leading to subsequent loosening and dislocation of the fracture plate, rupture [7], and bone resorption as a result of stress shielding [5]. Consequently, there exists a clear need for an alternative treatment which addresses these issues.

An alternative approach to affix maxillofacial plates is the use of a bone adhesive. This option presents advantages when compared to the use of screws [8]: The larger contact area of the adhesive ensures that the load is spread evenly over the bone-plate surface area, thereby avoiding stress concentration and subsequent fixation failure. In addition, adhesive fixation would negate the need for pilot holes, an advantage when attaching plates to weak bone or small bone fragments. The ideal properties for a successful bone adhesive include: chemical interaction with the surface of the bone, biocompatibility, and high bonding strength in the presence of body fluids [9,10,11]. Two main areas of research have been pursued in developing bone adhesives, namely adhesives that can be used to attach the IFP to bone and adhesives that can be used to attach the bone fragments together at the bone-bone interface [8]. Both areas have yet to produce a commercially available product, often due to the difficulty in maintaining a high bond strength in an aqueous environment. In dentistry however, a similar problem has been largely overcome with materials that can bond to dentine, a tissue that is chemically very similar to cortical bone [12], and which also requires bonding in a moist environment [13]. Consequently, a number of groups have looked at using difunctional methacrylate monomers as bone adhesives [14,15,16,17,18] with bond strengths reported greater than that found when titanium alloy IFPs are attached to bone through screw fixation [11]. However, unlike titanium alloys there is no inherent chemical interaction between the methacrylates and resorbable polymers, meaning that bond strengths are likely to be lower [11]. Consequently, we have developed a methacrylate-terminated resorbable copolymer suitable for use as an IFP, with the aim of producing a strong bond between IFP and dimethacrylate adhesives [19]. In this present work we describe the development of a model adhesive designed to provide a strong bond between this resorbable copolymer and cortical bone.

The process of bonding to dentine traditionally involves etching with an acid, followed by subsequent application of an amphiphilic monomer, known as a primer, before applying the bonding resin monomers. The amphiphilic properties of the primers allow the simultaneous wetting of hydrophilic collagen fibrils found within the dentine and interaction with the often-hydrophobic monomers used in bonding agents [13]. With progression in the field of adhesives, these steps have now been integrated with either the acid and primer combined, primer and bonding monomers combined or all steps combined in so-called one step products. However, throughout all these developments the importance of the priming agent to enable the interaction between the hydrophilic collagen network of the dentine and the hydrophobic bonding monomer remains pivotal.

**Figure 1:**
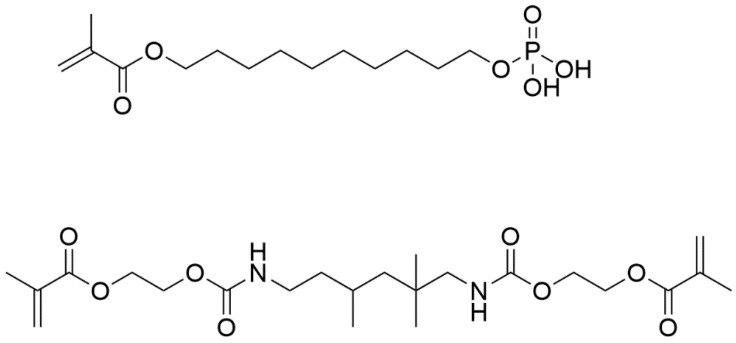
Chemical structures of MDP (**top**) and UDMA (**bottom**).

In this current research we investigate the use of the dental adhesive monomers, urethane dimethacrylate (UDMA), 10-methacryloyloxydecyl dihydrogen phosphate (MDP), as bone adhesives alongside an MDP-based priming agent (Figure 1). UDMA is a hydrophobic dimethacrylate monomer capable of forming densely cross-linked polymers [20], with high polymerisation rates and mechanical properties in the resulting polymer network [21,22]. However, UDMA does not form any chemical interactions with dentine, meaning that on its own it is unlikely to be a suitable bone adhesive. Monomers with functional groups capable of releasing one or more protons, such as carboxyl, phosphate, and phosphonate groups have been shown to chemically bind to calcium within hydroxyapatite [23]. MDP, a self-etching functional monomer has shown capacity to ionically bond to dentine [24] and has been noted to be the best commercially available functional monomer within current dental adhesives [25,26,27,28]. MDP has a polar diprotic acidic phosphate functionality and has been shown to interact intensively with hydroxyapatite [23,29]. In addition to its phosphate functionality, MDP has a non-polar saturated carbon chain which affords an amphiphilic nature, previously shown to equip the molecule to form interactions with collagen found in dentine [30], and implying that MDP also has the potential to be used as a primer to modify a hydrophilic bone/hydrophobic adhesive monomer interface. MDP is also relatively stable to hydrolysis and is durable in dental oral environments [31,32,33]. 

This study seeks to assess the use of MDP and UDMA as bone adhesives to affix IFPs to porcine mandibular bone in vitro. The chemical interactions of MDP and UDMA with bone were assessed using XRD and imaged using TEM. TEM and XRD revealed the formation of nano-layered structures with the MDP primer, a finding which to the best of our knowledge has not been reported on bone. The stability of the adhesives in physiologically relevant media was assessed over a six-week time period. The highest initial bond strengths were measured when an MDP primer was used prior to applying the copolymer of MDP and UDMA (termed MDP + U + P). Even without the application of the priming step, the bond strengths measured for the copolymer (MDP + U) were significantly higher than the lower threshold for successful bone adhesives of 0.2 GPa identified by Weber and Chapman [34]. Cytocompatibility of the formulations was determined using in vitro cell culture tests revealing that the MDP + U has an appropriate cytotoxicity profile for use as an adhesive to affix bio-resorbable IFPs.

## 2. Materials and Methods

### 2.1. Preparations of Adhesive Formulations and Primers

Two adhesive formulations were prepared. The first contained MDP mixed with 5 mol% deionised distilled water (nominal resistivity 18 MΩ cm, Nanopure™ purification system, Barnstead). The second combined UDMA and MDP (MDP + U) and containing 25 mM UDMA (Sigma Aldrich, Dorset, UK) and 1.25 mM MDP (Matrix Scientific, Elgin, SC, USA). Each formulation contained 1 mol% camphorquinone and ethyl-dimethyl aminobenzoate (Sigma Aldrich, Dorset, UK) and was stirred for a minimum of 30 minutes prior to use. A primer, termed formulation P, was prepared with 30 mol% MDP, 10 mol% distilled water and 60 mol% absolute ethanol (Fisher Scientific, Loughborough, UK). Bone samples were assigned to three different groups according to the adhesive used: MDP, MDP + U, and adhesive used with a priming step, MDP + U + P.

### 2.2. Preparation of Bone Samples and Polymer Discs

Porcine mandibles were obtained from a local butcher and disc specimens 10 mm in diameter were cut using a pillar drill (AJBM16, Ajax Machine Tools International Ltd., Hampshire, UK) and plug cutter. Next the discs were set in two-part polyester resin (UN1866, Easy Composites Ltd., Staffordshire, UK) with the cortical plate uppermost and polished under water using P600 and P1200 grit abrasive paper (3M, Bracknell, UK) to obtain a flat surface. Cut specimens were stored in 1 wt.% aqueous chloramine-T solution (Sigma Aldrich, Dorset, UK) and stored at 4 °C until use.

Hydroxyethyl methacrylate-terminated poly(lactic-co-glycolic acid) (HT-PLGA) was synthesised by ring opening polymerisation of lactide and glycolide as per [19]. Polymer discs were compression moulded (70 °C, 90 min 0.18 MPa) to yield discs of 8 mm diameter × 3 mm thickness for shear bond tests and 8 mm diameter × 1 mm thickness for cytotoxicity tests.

### 2.3. Preparation of Polymer Adhered Bone Samples and Shear Tests

Bone samples were assigned to three different groups according to the adhesive used: MDP, MDP + U, and MDP + U + P. For formulations that did not use a primer, adhesive was painted onto the bone surface, the HT-PLGA disc was placed on top and the adhesive was cured using a LED light curing unit (470 nm, Coltolux LED, Coltene Whaledent Ltd., Burgess Hill, UK) with a light intensity of 500 mW/cm^2^ (measure with a Bluephase Meter II Radiometer, Ivoclar Vivadent, Schaan, Liechtenstein). The light was applied for four cycles of 40 s at 90° angles (160 s total light application time). For formulations utilising primer, the primer was painted onto the bone using continuous brushing (15 s), dried using compressed air (15 s) and the adhesive formulation applied to the primed bone surface, the HT-PLGA disc was placed and the adhesive cured.

### 2.4. Shear Bond Strength Tests

Polymer-adhered bone samples were prepared, placed into 1 wt% aqueous chloramine T solution (300 mL, Sigma Aldrich, Dorset, UK) and stored at 37 °C. Samples were removed at time points of one, two and six weeks. Upon removal, shear bond tests were performed. Shear bond tests were performed with a universal testing machine (5567 Instron, Berks, UK). Specimens were placed in an in-house manufactured sharp guillotine test jig so that the guillotine blade met the interface between the bone and the HEMA-terminated PLGA plate. All tests were conducted at 0.5 mm/min crosshead speed and the shear bond strength was calculated as the maximum compressive force dived by the cross-sectional area. A sample size of five specimens was chosen for each group. Data obtained was analysed using a one-way ANOVA with a post hoc Tukey’s test (v19.1.1, Minitab Ltd., Coventry, UK).

### 2.5. Transmission Electron Microscopy (TEM)

Bone sections were demineralised and fixed simultaneously in a 10% formaldehyde-formic acid solution (Surgipath Decalcifier I) for 36 h. Two pieces of bone were used per adhesive system and fixed overnight in 2.5% glutaraldehyde in 0.1 M sodium cacodylate buffer (Agar Scientific, Stansted, UK). The samples were then post-fixed in 1% osmium tetroxide (TAAB Laboratories Equipment Ltd., Berks, UK) and subsequently dehydrated in an increasing series of acetone/water solutions (25 up to 100%) before being impregnated with epoxy resin. The resin blocks were then polymerised at 60 °C. Ultrathin sections (70–90 nm) were cut perpendicular to the adhesive bone-interface, stained with uranyl acetate and lead citrate (Leica Biosystems, Newcastle-upon-Tyne, UK) and then viewed on a TEM, (CM100, Phillips, Eindhoven, The Netherlands).

### 2.6. X-ray Diffraction (XRD)

Bone samples were coated in adhesive, cured (excluding the primer solution) and rinsed with distilled water. The crystal phases of the samples were identified using an X-ray powder diffractometer (PANalytical X’Pert Pro MPD, Philips PW3040/60 X-ray generator, X’Celerator* detector, Newcastle, UK), operated at 40 kV acceleration and 40 mA current, the data were collected over a range of 2–70° 2θ with a step size of 0.033° 2θ and nominal counting time per step of 250 s.

### 2.7. Cytotoxicity Testing

MC3T3 mouse osteoblasts (Sigma Aldrich, Dorset) were grown as monolayer cultures in T-75 flasks (Costar/Corning, Cambridge) using DMEM supplemented with 10% foetal bovine serum, 1% penicillin streptomycin and 1% L-glutamine (Gibco, Fisher Scientific, Loughborough, UK). The cells were subcultured three times per week and kept at 37 °C in an atmosphere containing 5% CO_2_ in air and 100% relative humidity. Cells were maintained at low passage number (5–18).

#### 2.7.1. Indirect Cytotoxicity Assay

Adherent cells at a logarithmic growth phase were detached by the addition of 1.5 mL trypsin, seeded at a density of 5000/200 µL of culture medium in 96 well plates and left for 24 h to proliferate.

50 µL of each adhesive system was placed between two HT-PLGA discs, sandwiched together and polymerised (160 s), HT-PLGA discs with no adhesive were used as a control. DMEM (5 mL per vial) was added and left for one, three or seven days. The extracted medium was then filtered through a 0.22 µm filter.

After 24 h cell growth in the 96 well plates, 200 µL of extract medium was added and cells were incubated for a further 24 h, control wells were treated with 200 µL of DMEM. Six replicate wells for each adhesive were prepared. At the indicated time, cell viability was estimated by means of an MTT 3-(4,5-dimethylthiazol-2-yl)-2,5-diphenyltetrazoliumbromid) assay. The culture medium was aspirated prior to washing with PBS (200 µL) and addition of MTT reagent (Sigma Aldrich, Dorset) (200 µL 1 mg/mL in DMEM). Microplates were left for 4 h then cells were lysed using DMSO (100 µL for 30 min) before being read spectrophotometrically (570 nm, reference wavelength 650 nm). Cell viability was calculated as a percentage normalised to the control wells.

#### 2.7.2. Direct Cytotoxicity Assays

A direct contact method was performed according to ISO 10993. Prior to testing two HT-PLGA discs were sandwiched together using the adhesive formulations and cured (n = 3 for each formulation). The sandwiched discs were sterilised using UV light (254 nm, 40 min each side) and secured to the bottom of a 24 well-plate using sterilised vacuum grease. Adherent cells at a logarithmic growth phase were detached by the addition of 1.5 mL trypsin, and seeded onto the sandwiched discs at a density of 2 × 10^4^ cells/ml of culture medium. The cells were incubated for one, three and seven days, the polymer discs were removed, and the cell viability evaluated using an MTT assay.

## 3. Results

### 3.1. In Vitro Artificial Aging

**Figure 2:**
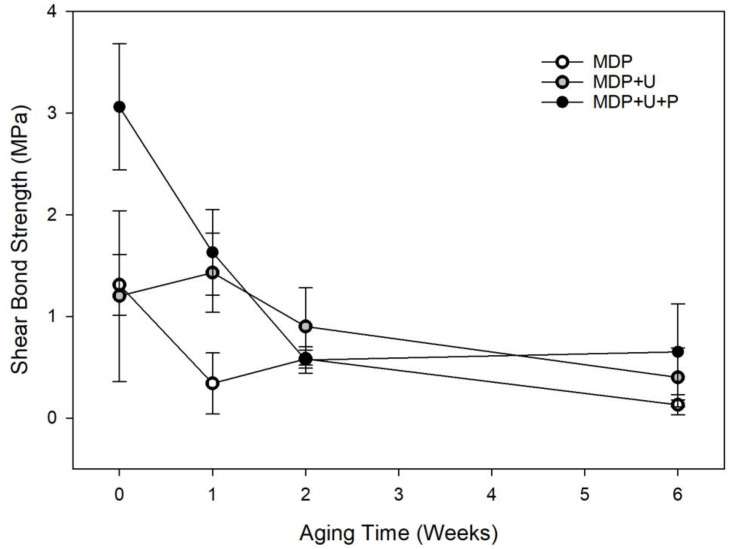
Shear strength of adhesive formulations during 6-week artificial aging study. The symbols represent the mean shear bond strength with error bars representing the SD.

The shear bond strength significantly reduced over the aging period irrespective of choice of adhesive (*p* < 0.001). When controlling for aging period adhesive choice significantly affected shear bond strength (*p* < 0.001). The addition of UDMA to MDP improved the bond strength up to week 1. This was further improved when a priming stage was added, Figure 2.

### 3.2. TEM

**Figure 3:**
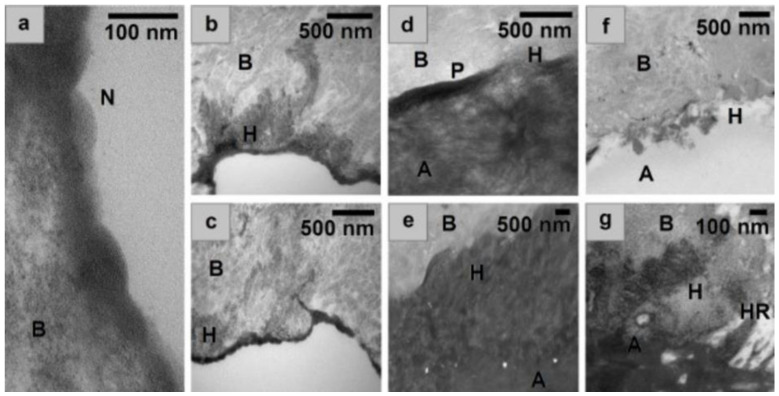
(**a**) nanolayering exhibited in the surface of the bone by the MDP primer, (**b**,**c**) MDP primer, showing integration of primer into the collagen network of the bone. Spacing of layers is equal to ~3.5 nm, (**d**) MDP + U + P, (**e**) MDP, (**f**) MDP + U, (**g**) expansion of e to showing integration, with a resin tag in d, as adhesive penetrates an open pore. B = Underlying bone demineralised during TEM processing, H = Hybrid layer N = Nanolayering, HR = Phenomenon similar to hybrid resin tag, P = Primer layer, A = Adhesive.

Results The MDP primer exhibited nano-layering on the surface of bone with spacing of layers equal to ~3.5 nm (Figure 3a). This spacing correlated with photomicrographs obtained of MDP on dentine and hydroxyapatite samples [35]. The primer also exhibited integration into the collagen network of the bone substrate forming hybrid layers (Figure 3b,c). The representative photomicrographs illustrate that both the MDP and UDMA adhesive formulations formed hybrid layers with the underlying bone (Figure 3d–f). MDP exhibited a deep hybrid layer (~3.5 µm) (Figure 3b–e), in comparison the MDP + U adhesive which had a much shallower (~400 nm) less defined hybrid layer (Figure 3f). Photomicrographs of MDP + U + P (Figure 3d) show an electron dense area at the surface of the bone attributed to the primer hybrid layer under the polymerised UDMA.

### 3.3. XRD

**Figure 4:**
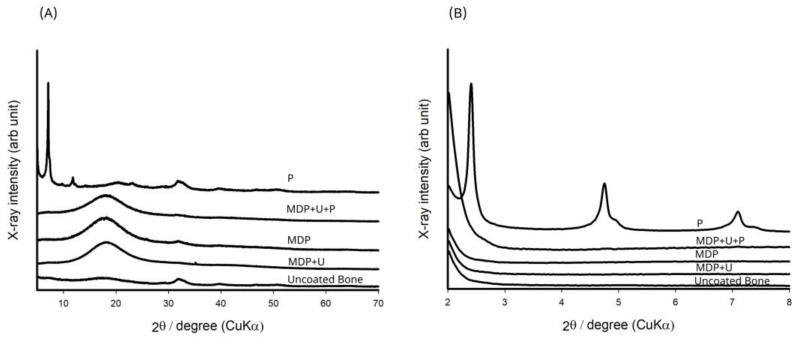
(**A**) XRD analysis of the adhesive monomers interacting with the bone substrate. (**B**) Magnified image of the area between 2° and 8° in (**A**) revealing interactions of the primer layer (P) with bone.

Uncoated bone samples were compared with the XRD database (01-074-9762, Ca 4.938 (PO_4_)_3_OH 0.810 calcium phosphate hydroxide, hydroxyapatite, Q:S:Hexagonal, ICDD Powder Diffraction File Databases (1999, 2004, accessed 6 November 2014)), and were found to be well matched. Figure 4 shows resonances at 11.4° (d = 0.78 nm) and 23° (d = 0.38); these have been ascribed to CaHPO_4_·2H_2_O as a reaction product [35,36]. The 10-MDP primer revealed three characteristic sharp resonances at 2θ, 2.4° (d = 3.67), 4.7° (d = 1.86), 7.1° (d = 1.24) and some broad peaks at 20.1°, 31.7° and 39.7°. As previously reported by Yoshihara et al. [35] and Fukegawa et al. [36] these resonances indicate the presence of a crystalline phase with a nano-layered structure that has d spacing of 3.67, 1.86, and 1.24 nm. In comparison, the cured adhesive formulations did not reveal any distinguishing resonances.

### 3.4. Cytotoxicity Assays

**Figure 5:**
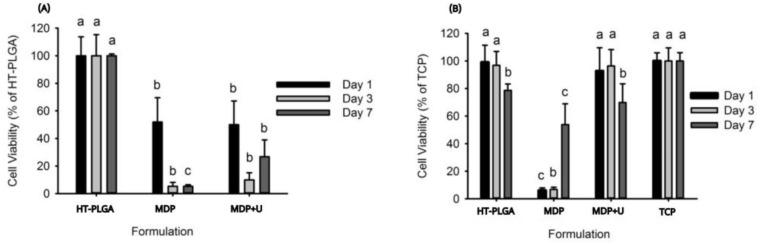
(**A**) Direct cytotoxicity assay, (**B**) Indirect cytotoxicity assay, Tissue culture plastic (TCP). Lowercase letters (a,b,c) denote statistical groupings at each time point.

The direct MTT assays conducted on HT-PLGA discs sandwiched with adhesive formulations showed the adhesives to be cytotoxic with viability less than 50% throughout (Figure 5A). The indirect MTT showed that UDMA obtained the same statistical grouping as the HT-PLGA discs (Figure 5B). The MDP formulation improved with time achieving 60% viability after day 7.

## 4. Discussion

While it is difficult to put a specific value on the bond strength required from a bone adhesive, Weber and Chapman [34] identified a lower threshold value of 0.2 MPa, below which it is difficult to maintain fracture reduction. In order to attach bio-resorbable IFPs this bond strength must be maintained under physiologically relevant conditions for a period of time that is long enough for the fracture to heal sufficiently, usually six weeks. The first experiments established the effect of artificial aging at 37 °C in chloramine T solution upon the shear bond strength (SBS) of the adhesive formulations (Figure 2). The SBS of the three monomer formulations were initially found to be between 1.4 and 3 MPa. MDP + U + P showed significantly higher SBS, yielding values over double those obtained by the other two formulations demonstrating that the amphiphilic primer is able to modify the interface between the adhesive and bone resulting in higher initial bond strengths. After one week MDP + U + P presented no significant effect on SBS, suggesting that the priming step successfully improves initial bonding of the adhesive formulations to bone, having no subsequent detrimental effects on SBS of the adhesive in wet conditions. This finding is consistent with previous research that showed that MDP can potentially improve bond durability after long-term water storage [37], with MDP containing adhesives producing increased microtensile bond strengths to superficial dentine during nine months water storage experiments [38].

In the present study, after two weeks in aqueous storage there were no statistically significant differences between the formulations tested. With the exception of formulation MDP at six weeks, all the bond strengths achieved throughout the experiment are significantly higher than those identified by Weber and Chapman, thus the adhesive formulations have sufficient SBS to be used in principle to affix bio-resorbable IFPs. We hypothesise that the lower bonding strengths achieved by the MDP formulation are on account of the instant curing of the MDP adhesive, this reduced the time for the formulation to infiltrate into the bone prior to curing. It has previously been shown that utilising a scrubbing technique when applying dentine adhesives facilitates the evaporation of solvent, enabling higher levels of monomer impregnation and the development of a better adhesive interface [39]. Consequently, when the primer was applied, a continuous brushing (15 s) technique was used to ensure monomer diffusion into the substrate [40]. This allowed the MDP primer to etch the surface and infiltrate the bone more efficiently. UDMA is also known to form densely cross-linked polymers [20], a contributing factor in the longevity of its adhesion. The bond strengths obtained are comparable to those obtained by Kandalam et al. [11] who studied the use of novel and commercially available cyanoacrylate and methyl methacrylate adhesive formulations to bond commercially available poly L-lactic acid poly glycolic acid bio-resorbable plates (Lactosorb Biomet Micro-fixation) to bone.

The photomicrographs of the primer/bone interfaces revealed the formation of nano-layered structures with spacing of approximately 3.5 nm (Figure 3a). These nanolayers have been previously reported in TEM and STEM-EDS analysis of substrates of hydroxyapatite and dentine treated with MDP [29,35]. When MDP calcium salts were analysed, nanolayers of 3.51 nm were reported, similar to those found in the present study [41]. To form these nanolayers the MDP molecules arrange themselves with their methacrylate groups directed towards each other, and their phosphate groups in the opposing direction, with deposition of calcium salts between the layers [24,29,35]. The hydrophobic spacer between the phosphate and methacrylate functional groups has been shown to allow the MDP to interact with collagen found in dentine [30] allowing improved diffusion of the adhesive into the dentine [32], producing a better hybrid layer that that found with non-MDP containing commercially available dentine adhesives [42]. The similarity in the nanolayers found in this present study with those shown previously for dentine lead us to conclude that MDP must have the same action when used as a bone adhesive.

When the adhesives were used without a primer only a very shallow and non-uniform hybrid layer was formed, likely on account of the opposing hydrophilicities of the UDMA and bone. In contrast, when the primer was used an electron dense layer was observed below the UDMA. These findings are reflected in the initial SBS of the formulations, where the primer had a significant positive effect, increasing the SBS of the MDP + U formulation. This improvement did not last throughout the whole aging period, something previously reported for MDP-treated dentine samples stored in water for up to a year [43]. With the MDP formulation a hybrid layer up to a thickness of 3 µm was observed (Figure 3b). Previous work [44] has reported mild self-etch monomers attain hybrid layers of only 0.5–1 µm depth on dentine substrates. This increased hybrid layer depth found in our study probably exists due to the high concentration of the acidic MDP moiety within our formulation, which is likely to have contributed to the greater penetration depth. Additionally, it is possible that by sanding our bone specimens prior to use we may have increased the porosity of the underlying bone.

The XRD results for MDP suggest the formation of a crystalline layered structure with a molecular orientation where the acidic phosphate moieties interact with the bone, and the saturated carbon chains align themselves back-to-back. Fukegawa et al. [36] first proposed this mechanism of MDP absorption onto hydroxyapatite using XRD analysis to assess the chemical interactions of phosphoric acid esters with hydroxyapatite. Shoulder resonances observed alongside the resonances at 4.7° (d = 1.86), 7.1° (d = 1.24) indicate the presence of both the mono and di-calcium salt of the MDP dimer [26]. It was not possible to see any crystalline phases upon treatment with the monomer formulations, probably on account of the amorphous nature of the cured polymers.

The results of the indirect cell contact assay suggest that when MDP is formulated with UDMA it has an appropriate cytotoxicity profile with the potential to be used as an adhesive to affix bio-resorbable IFPs. While the all the monomers are cytotoxic, previous work has established that extracted media which has been exposed to cured commercially available dental adhesives, including one containing MDP, had only a minor effect when incubated with fibroblastic cell lines [45]. We propose that the cytotoxicity profile of MDP is most likely caused by its acidic nature (p*K_a_* 1.97, 6.82 [46]) and we have shown that when MDP is formulated with UDMA minor toxicity effects are observed from the leachable components.

The results of the direct cell contact assay correlate with previous cytotoxicity studies of dental agents [47] that assessed the direct cytotoxicity of commercially available primers and bonding agents towards human pulp derived cells. All of the adhesives disturbed the cellular redox state of pulp cells in monolayer cultures, however, despite their low biocompatibility in direct contact with cells, these formulations are currently being used in clinic as dental adhesives. Though the direct cytotoxicity profiles of UDMA and MDP are unfavourable, the desired application of these adhesive formulations is to affix bio-resorbable IFPs to bone, therefore they are not required to be bioactive or designed to be applied onto soft tissues. The indirect cytotoxicity assay provides a toxicity profile of the adhesive formulations that is more viable for the application specified, and the results from this assay conclude that MDP has an appropriate cytotoxicity profile when used in conjunction with UDMA.

## 5. Conclusions

Two formulations, together with a priming step, were assessed for their ability to affix bio-resorbable IFP’s to bone. In a six-week artificial aging study both MDP and MDP + U achieved SBS greater than the lower threshold identified by Weber and Chapman [34] indicating the adhesive formulations have sufficient SBS to be used to affix bio-resorbable IFPs. The addition of a priming step increased the initial bond strength but this increased strength did not last after 2 weeks, by which stage there was no significant difference with the bond strength of the MDP + U specimens. Further analysis by TEM revealed that the MDP primer produced a crystalline nano-layered structure with spacing of ~3.5 nm on bone. XRD revealed chemical interactions between the adhesive formulations and bone in an orientation consistent with that proposed using hydroxyapatite as a substrate [29,36]. The cytotoxicity profiles of the adhesive formulations were determined using indirect and direct contact with MC3T3 cells, with indirect conditions suggesting MDP + U is as biocompatible as HT-PLGA. Due to its cytotoxicity profile MDP alone is not a viable adhesive for use to affix bio-resorbable IFP’s, however when used in conjunction with UDMA, MDP can be part of potential bone adhesive formulations. We believe that these data show the potential of MDP copolymers for use as bone adhesives, with the formation of the hydroxyapatite nanolayer suggesting that these adhesives could form a hybrid layer with the bone, potentially leading to the development of strong adhesion, sufficient to attach IFPs to bones without the need of screws.

## Data Availability

The data presented in this study are openly available at 10.25405/data.ncl.21640454.
